# Optical Dynamics of Copper-Doped Cadmium Sulfide (CdS) and Zinc Sulfide (ZnS) Quantum-Dots Core/Shell Nanocrystals

**DOI:** 10.3390/nano12132277

**Published:** 2022-07-01

**Authors:** Muhammad Haroon Rashid, Ants Koel, Toomas Rang, Nadeem Nasir, Nadeem Sabir, Faheem Ameen, Abher Rasheed

**Affiliations:** 1 Department of Textile Engineering, National Textile University, Faisalabad 37610, Pakistan; haroonrashid@ntu.edu.pk (M.H.R.); abher.rasheed@ntu.edu.pk (A.R.); 2 Thomas Johann Seebeck Department of Electronics, Tallinn University of Technology, Ehitajate tee 5, 12616 Tallinn, Estonia; ants.koel@ttu.ee (A.K.); toomas.rang@ttu.ee (T.R.); 3 Department of Applied Sciences, National Textile University, Faisalabad 37610, Pakistan; nadeemnasir@ntu.edu.pk; 4 Department of Physics, Government College University, Faisalabad 38000, Pakistan; 5 School of Natural Sciences, National University of Sciences and Technology, Islamabad 44000, Pakistan; faheem.amin@sns.nust.edu.pk

**Keywords:** quantum dots (QDs), nanocrystals, copper doping, cadmium sulfide, zinc sulfide, optical properties, photoluminescence

## Abstract

Recently, quantum-dot-based core/shell structures have gained significance due to their optical, optoelectronic, and magnetic attributes. Controlling the fluorescence lifetime of QDs shells is imperative for various applications, including light-emitting diodes and single-photon sources. In this work, novel Cu-doped CdS/ZnS shell structures were developed to enhance the photoluminescence properties. The objective was to materialize the Cu-doped CdS/ZnS shells by the adaptation of a two-stage high-temperature doping technique. The developed nanostructures were examined with relevant characterization techniques such as transmission electron microscopy (TEM) and ultraviolet–visible (UV–vis) emission/absorption spectroscopy. Studying fluorescence, we witnessed a sharp emission peak at a wavelength of 440 nm and another emission peak at a wavelength of 620 nm, related to the fabricated Cu-doped CdS/ZnS core/shell QDs. Our experimental results revealed that Cu-doped ZnS shells adopted the crystal structure of CdS due to its larger bandgap. Consequently, this minimized lattice mismatch and offered better passivation to any surface defects, resulting in increased photoluminescence. Our developed core/shells are highly appropriate for the development of efficient light-emitting diodes.

## 1. Introduction

Quantum dots (QDs) are pint-sized materials [1] that exhibit unique and exceptional optical and electronic properties compared with those of bulk materials. QDs are often referred to as zero-dimension crystal structures due to their extremely small size. Generally, the diameter of QDs stays in the range of 2–10 nm, which is smaller or closer to the dimension of the Bohr exciton radius [2]. QDs are considered a replacement for traditional organic dyes or fluorescent proteins [3] as they suffer less from photo-bleaching in comparison with their entrants. The optoelectronic properties of QDs show interesting phenomena, such as size-dependent narrow peak emission, broad excitation range, strong absorption, high chemical stability, and a limited number of electrons compared with organic dyes or fluorescent proteins [4]. Because of these properties, QDs are prime candidates for various professional and commercial applications including solar cells, bio-labeling, electronics, optical detection of biomolecules, and, more recently, QD lasers [5,6,7,8,9,10,11,12,13,14,15].

The photoluminescence properties of QDs can be tuned accurately with sharp emission depending on their composition and size [16,17,18,19,20,21,22,23,24,25,26,27,28,29,30]. Furthermore, single QDs have a high surface-to-volume ratio catering to a significant number of surface trap states, which then act as de-excitation (non-radiative) centers for photo-generated charge carriers. The trap states play a pivotal role in optoelectronic devices because such devices rely on the relaxation time of charge carriers. The trap states result in the reduction in the fluorescence efficiency of the quantum dots due to the Auger recombination. To recover the photoluminescence of QDs, a shell of suitable thickness is grown on the surface of the QDs. The core/shell QDs are more robust against environmental changes, surface chemistry, and photo-oxidation [31,32]. The core/shell structures are typically classified as type-I or type-II based on the alignment of the energy bandgaps between valence and conduction bands [33]. In type-I materials, the shell, having a larger bandgap than the core, provides a physical barrier to the charge carriers and helps in surface passivation and carrier confinement within the core. Typically, core/shell structures are composed of II-VI, IV-VI, and III-V group semiconductors with suitable combinations such as CdS/ZnS, CdSe/CdS, and InAs/CdSe [34]. Group II-VI semiconductor QDs are very attractive materials because of their wider range of optical, optoelectronic, and magnetic properties. These materials exhibit metastable crystal structures, such as zinc blende and wurtzite [35,36], providing strong exciton–photon interaction with a wider range of transition energies.

Moreover, controlling the fluorescence lifetime of QDs is particularly important as it is a prerequisite to various applications, including light-emitting diodes, single-photon sources for quantum information, fluorescence resonance energy transfer (FRET), and enhancement in nearby fluorophores, to mention but a few. A prudent way of achieving a reasonably longer fluorescence lifetime is by introducing stable energy states in the bandgap of host materials. Numerous studies have been carried out to introduce transition metals into the host core/shell structures. The location of incorporated metal ions was found to exert a prominent effect on the properties of the crystals. The most effective region is the interface between the core and the shell of QDs, as energy remains at a minimal level at the interface. In the present study, we chose a type-I CdS/ZnS core/shell system and Cu as a dopant material. ZnS has a larger bandgap than the CdS core, and the crystalline structure of the ZnS shell adopts the crystal structure of the CdS core to minimize lattice mismatch, along with offering better passivation to surface defects, resulting in an increased photoluminescence efficiency. CdS/ZnS core/shell QDs are suitable for high luminescence intensity and chemical stability [37]. CdS and ZnS have direct bandgap energies of 2.9 and 4.8 eV, respectively, at room temperature [38]. Moreover, Cd^+2^ and Zn^+2^ have the same ionic charge and not too dissimilar radii. They can substitute each other in lattices, such as a sulfide crystal lattice (CdS and ZnS), without altering the crystal structure of the host material.

Furthermore, doped and undoped core/shell structures can be prepared through various methods such as phase precipitation [39], micro-emulsion [2], microwave irradiation [40], and two-step synthesis [41]. The two-step synthesis method is a convenient method to fabricate the CdS/ZnS structure because it provides effective control over size and size distributions [42]. The method has several advantages, such as easy handling, inexpensive raw materials, producing high-purity samples, and less agglomeration than is common in other methods [43]. Transition metals, such as Mn, Cu, Co, and Ni, are frequently used to dope different materials because they provide further opportunities to combine different optical and electronic properties into a single material, such as a core/shell structure [44]. The ionic radius of these metals is larger than that of Cd^+2^ and Zn^+2^. Therefore, they can easily displace Cd^+2^ and Zn^+2^ of the host lattice and occupy the interstitial site of the host lattice. When these metal ions are doped into the CdS QDs, Cd ions are replaced by Cu ions in the host lattice and, as a result, Cu deep centers are formed, which can capture more electrons and holes to be excited, enhancing the de-excitation processes [45,46,47,48]. We synthesized both undoped and Cu-doped core/shell QDs through a two-step synthesis method and characterized their structural and optical properties using ultraviolet–visible (UV–Vis) spectroscopy, photoluminescence spectroscopy (PL), and transmission electron microscopy (TEM).

## 2. Materials and Methods

The chemicals used in this study were Oleylamine (Sigma-Aldrich, St. Louis, MO, USA), sulfur powder (Sigma-Aldrich), 1-octadecence (Sigma-Aldrich), sodium diethyldithiocarbamatetrihydrate (Sigma-Aldrich), and zinc stearate (Carl Roth GmbH & Co., Karlsruhe, Germany), as well as Cu (II) acetate tetrahydrate (Thermo Fisher Company, Waltham, MA, USA).

### 2.1. Cadmium Sulfide (CdS) Core

**Figure 1:**
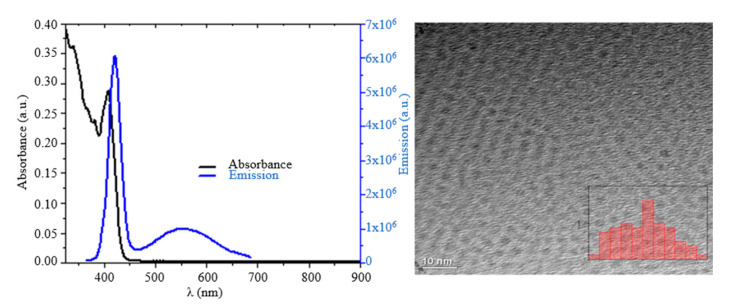
(**Left**) Absorption (black) and emission (blue) of CdS NPs. The sample was excited at a wavelength of 380 nm. The excitonic peak was 405 nm, as shown in the absorbance profile. (**Right**) TEM image of CdS NPs. The average diameter, shown by inset, of the CdS NP was 3.01 nm with σ = 0.23 (3.01 ± 0.23 nm).

Zinc blende CdS nanoparticles were prepared following previously published methods [48,49]. For this, 0.126 g CdO (0.98 mmol), 2.02 g oleic acid (7.1 mmol), and 12 mL ODE were added into a three-neck flask. The mixture was degassed for 10 min in the presence of N_2_ gas, and the temperature was raised to 300 °C. At this stage, 2 mL of S-ODE solution (0.25 M) was swiftly added to the flask, and the temperature was adjusted to 250 °C. The reaction was kept at this temperature for 5 min to allow for the growth of nanoparticles. The reaction was stopped by removing the heating mantle, and the solution was left to cool down at room temperature. Finally, the particles were washed by precipitating through acetone and methanol and resuspended into chloroform. Figure 1 depicts the average diameter of CdS nanoparticles taken with TEM (TEM 3010 JEOL Ltd., Tokyo, Japan) along with variability.

### 2.2. Copper-Doped Zinc Sulfide (ZnS) Shell

To incorporate Cu into the CdS core, CdS was dissolved in chloroform and added into a mixture of ODE and OAm (8.0 mL, ODE:OAm(3:1)) in a three-neck flask. The chloroform was removed from the solution under vacuum in the presence of N_2_ flow. The solution was heated to 250 °C, and 0.22 mL of Cu precursor was prepared following the method used in a previous study [50]. After, prepared precursor was added into the solution dropwise. The solution was then stirred at 250 °C for 20 min [50], followed by ZnS shell growth.

**Figure 2:**
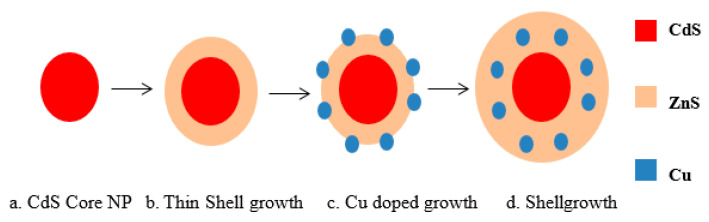
Schematic representation of Cu-doped ZnS and CdS shell.

Further to this, the ZnS shell was subsequently grown around the CdS core and Cu-doped CdS monolayer by substituting zinc stearate and sulfur solution in ODE. The sulfur precursor solution (40 mM) was prepared by dissolving the sulfur powder in ODE at room temperature, while the zinc precursor solution (40 mM) was prepared by dissolving zinc stearate in ODE at 130 °C until completely dissolved. The required quantity of the precursor solution of shell materials (Zn and S) was found by using the volume of the ZnS molecules [51]. The first monolayer, Zn, and S precursor solutions were added sequentially via strings to the reaction flask enclosing the CdS cores at 220 °C, waiting for 10 min after each addition. After the last Zn precursor injections, the temperature was maintained for 10 min and, finally, the reaction was stopped by removing the heating mantle and allowing the solution to cool. The resulting QDs were further cleaned by three precipitation–redispersion steps using chloroform and methanol. Finally, Cu-doped CdS/ZnS QDs were re-dispersed in toluene [52]. A schematic representation of the doping process is shown in Figure 2.

## 3. Results and Discussion

**Figure 3:**
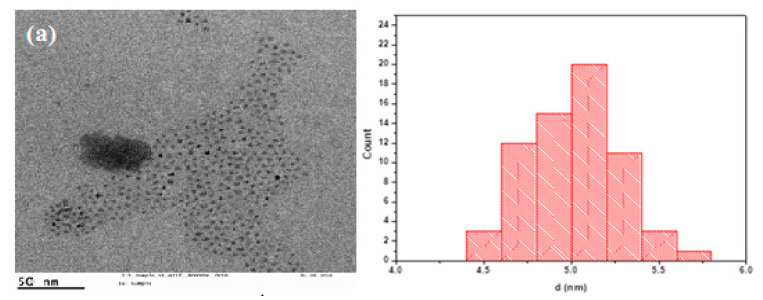

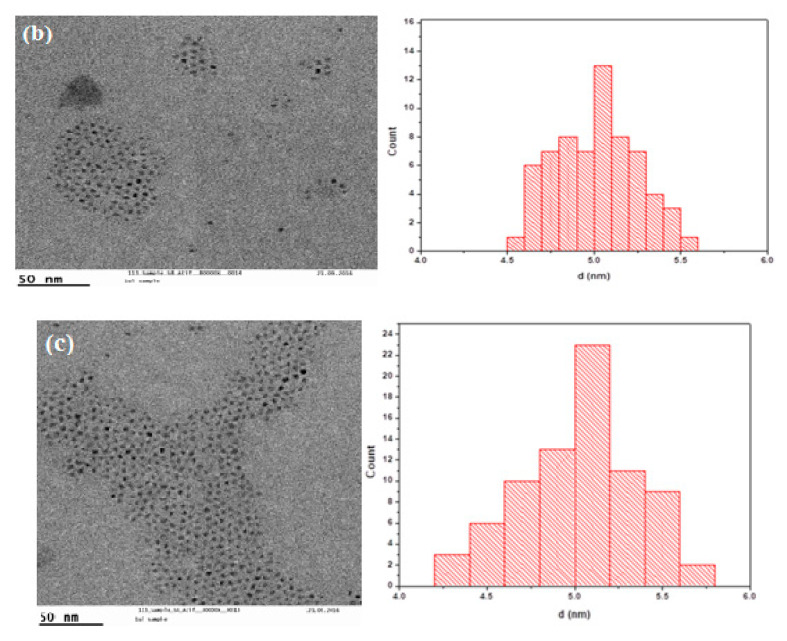
TEM analysis of doped CdS/ZnS quantum dots for different concentrations of Cu precursor. (**a**) Cu precursor of 0.78 µM, the an average diameter of the NPs was 4.99 nm with σ = 0.3 nm; (**b**) Cu precursor of 1.56 micro molar, the average diameter of the NPs was 5.01 nm with σ = 0.26; (**c**) Cu precursor of 3.12 micro molar, the average diameter of the NPs was 5.01 nm with σ = 0.33 nm.

This section provides a comprehensive discussion on the results that were achieved. Transmission electron micrographs of the undoped and doped CdS/ZnS core/shell nanoparticles are shown in Figure 3a–c, corresponding to their size distribution. The particles were found to be spherical, with an average diameter and narrow size distributions of 4.9 ± 0.3, 5.01 ± 0.26, and 5.01 ± 0.33.

**Figure 4:**
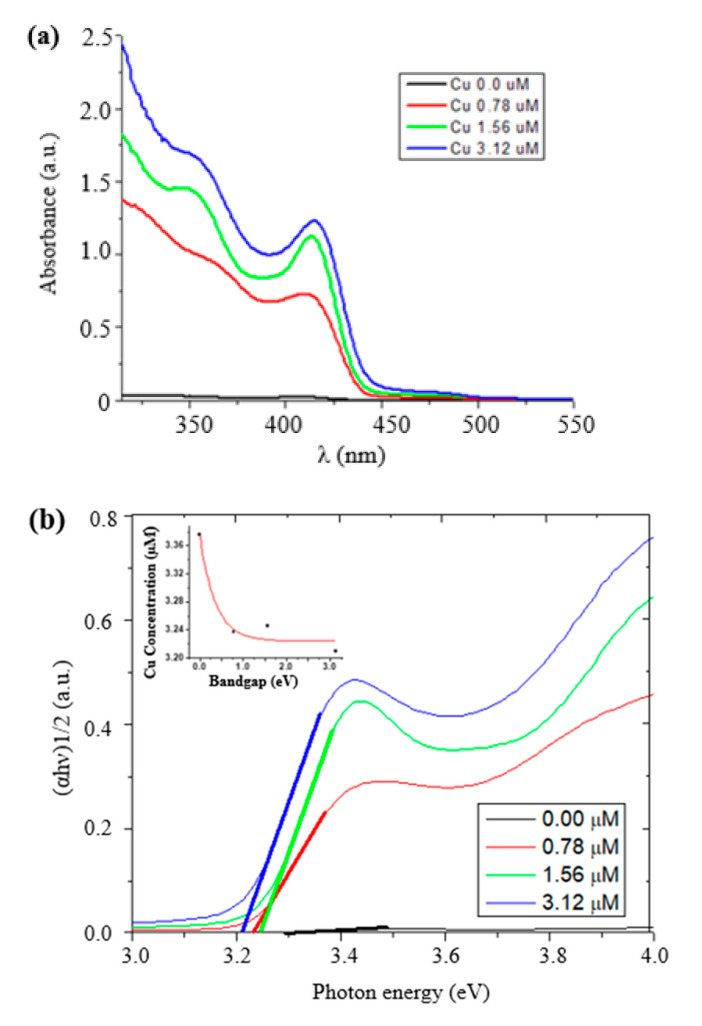

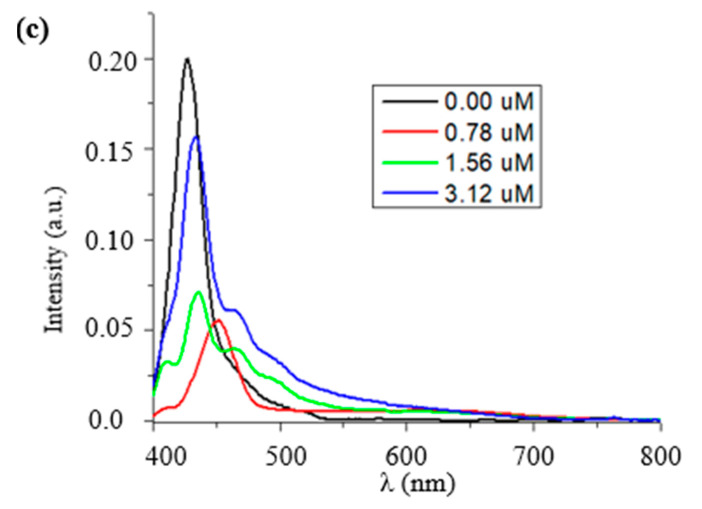
(**a**) UV–Vis spectra of doped and undoped CdS/ZnS core/shell nanoparticles; (**b**) bandgap calculation of all samples using Tauc plot; (**c**) Photoluminescence (PL) spectra of all samples (the photoluminescence intensity was found to initially decrease and then gradually increase with increasing concentration of Cu ions. The excitation peak was observed at a wavelength of 620 nm).

UV–Vis spectroscopy (Beckman Coulter, Brea, CA, USA) was performed to investigate the UV–Vis spectrum of the fabricated structures. UV–Vis absorption spectra belonged to the undoped and doped CdS/ZnS core/shell QDs, with varying doping concentrations, as depicted in Figure 4a. The edge of the absorption for doped CdS/ZnS core/shell QDs peaked at 406 nm and shifted toward the red region at different doping concentrations than those exhibited by the undoped CdS/ZnS QDs. This peak shift was an indication of the appearance of metastable trap states in the bandgap of pure material. At higher concentrations, the wavefunction of dopant electron orbitals started overlapping with the wavefunction of electrons in the conduction band. This resulted in the shift in the bandgap of the doped material. The bandgap of doped materials was calculated using the Tauc plot and is shown in Figure 4b. An inverse relationship was witnessed between bandgap and Cu concentrations that is there was decreased in bandgap and were noticed in association with increased Cu concentration.

**Figure 5:**
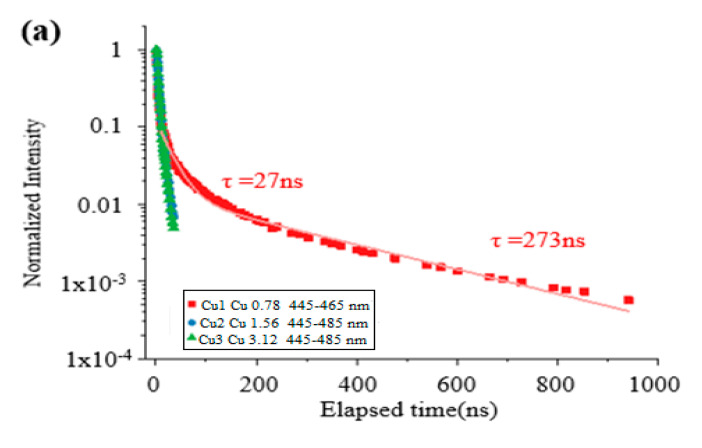

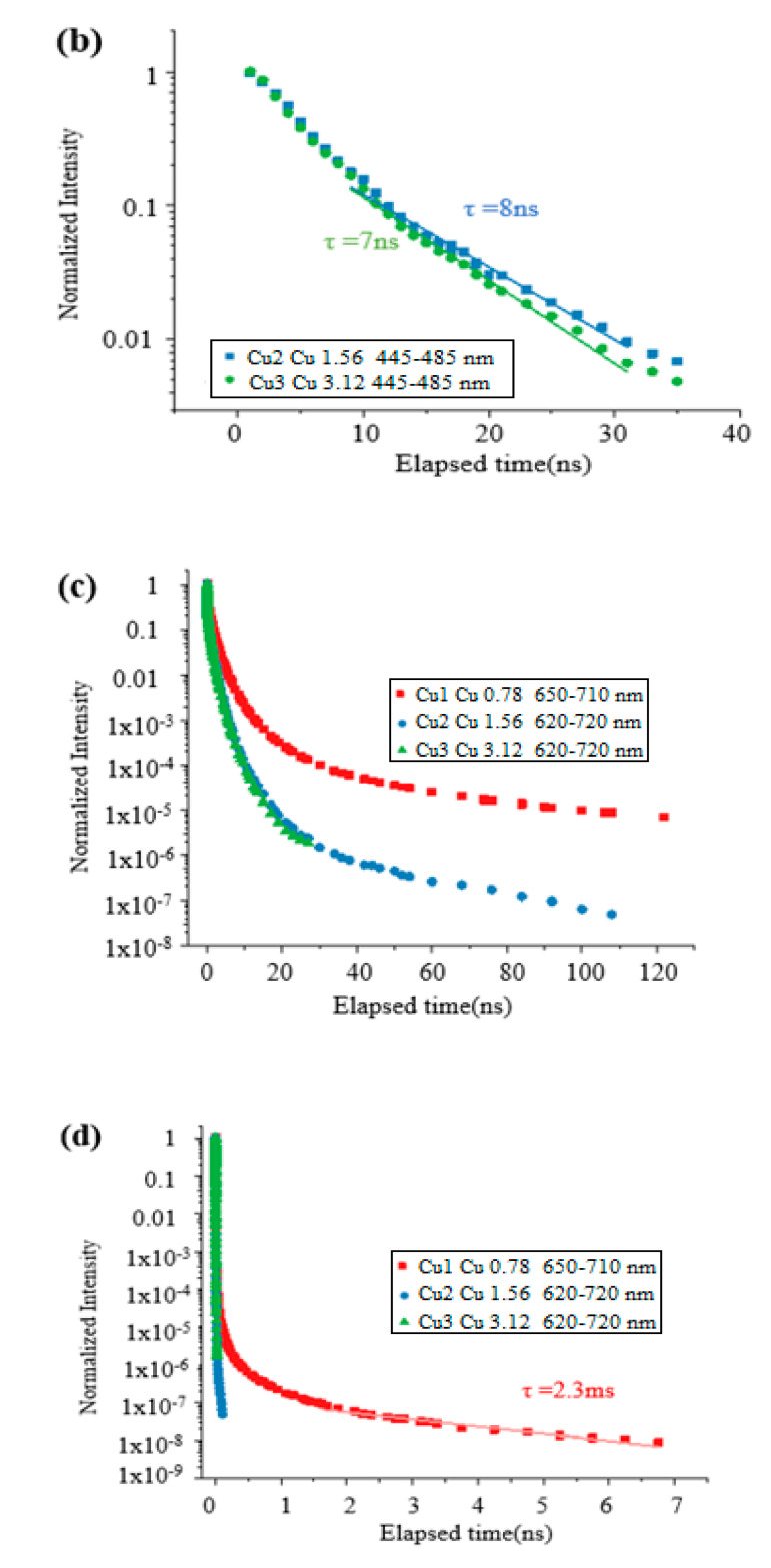
Decay of the higher energy PL (~460 nm) in Cu samples. Here (**a**) QD luminescence of sample Cu1 at 455 nm slower than luminescence band of sample Cu2 and Cu3 around 465 nm, (**b**) The broad luminescence around 465 nm, (**c**) The broad luminescence decay around 660 nm decay and (**d**) The broad luminescence decay around 670 nm decay.

Furthermore, photoluminescence spectroscopy (Spectra Physics Tsunami, CA, USA) was used to investigate the PL spectra of fabricated core/shell QDs. Figure 5 demonstrates the PL spectra of undoped and doped CdS/ZnS core/shell QDs for various pre-considered concentrations of doping. The emission peak of Cu-doped CdS/ZnS core/shell QDs was initially red-shifted in comparison with the undoped CdS/ZnS QDs. However, as the Cu concentration increased, the emission peak blue-shifted and exhibited increased intensity, contrary to other reports [3]. Furthermore, with increasing Cu concentration, new peaks were observed in PL spectra. 

This may be attributed to the migration of Cu atoms into the ZnS shell due to the preferential shelling of CdS to Zn compared with that of the CdS shell [3]. The time decay curves for different PL bands are shown in Figure 5. The PL spectra were recorded over a decay time between 10 and 30 ms. The decay spectra of the doped samples were divided into two regions: (i) short-wavelength (445–465 nm) and (ii) long-wavelength (650–710 nm). Furthermore, the overall transition states were divided into three regions: short- (10–20 ns), medium- (20–50 ns), and long-lived (50–125 ns) states. The decay rate (τ) for all the samples was approximately the same, which meant that the transition occurred between the same energy states during 10–20 ns. However, the decay dynamics were slower (τ = 273 ns) for Cu 0.78 (12-atoms) than for the Cu1.56 (24-atoms) and Cu3.12 (36-atoms) samples (τ = 27 ns) in the short-wavelength region. For the transition events in the long-wavelength region, the decay rates for the three samples were faster than the decay rates in the short-wavelength region (τ = 7–8 ns) for short-lived states, while the decay rates were much slower in the long-lived states (τ = 30 ms) for all the samples. The decay dynamics of the core/shell systems were divided into different time regions. In this case, four different regions were chosen (data not shown). In a time-domain between 10 and 30 ms, both short- and long-lived PL signals were visible. In a time-domain between 10 and 20 ns, only short-lived PL signals dominated the spectra. In the time domain between 20 and 50 ns, and between 50 and 125 ns, however, PL signals with a significantly slower decay time than the laser were still visible. The decay curves were fitted mono- or biexponentially to calculate the decay times of different curves.

The narrow QD luminescence of sample Cu 0.78 µM (12-atoms) at 455 nm decayed slower than the broader luminescence band of samples Cu1.56 µM (24 atoms) and Cu 3.12 µM (36 atoms), around 465 nm. A biexponential fit of the decay curve of the Cu (0.78 µM) luminescence for the first 1000 ns resulted in a short lifetime of approximately 27 ns and a long lifetime of around 273 ns, as shown in Figure 5a. The broad luminescence around 465 nm decayed similarly fast in samples two and three. A mono-exponential fit led to a decay time of around 7–8 ns, as shown in Figure 5b. The broad PL around 660 nm decayed faster in samples Cu2 and Cu3 than in sample Cu1. It decayed similarly in samples Cu2 and Cu3, but the PL was observed to be longer in sample Cu2 because of the higher intensity. The broad PL around 670 nm in sample Cu1 was observed up to several milliseconds. A mono-exponential fit suggested a long decay time of 2.3 ms, as shown in Figure 5d.

## 4. Conclusions

In this study, we synthesized novel copper-doped CdS/ZnS QDs with the application of a one-pot synthesis method. The copper-doped nanocrystals were further characterized using photoluminescence (PL) spectroscopy, transmission electron microscopy (TEM), and ultraviolet–visible (UV–Vis) spectroscopy. The Cu-doped CdS/ZnS QDs showed two peaks; the sharp emission peak was due to CdS QDs at 450 nm, while the broad emission peak (620 nm) was due to typical Cu ions, differing from the undoped CdS/ZnS nanocrystals. The photoluminescence properties of our developed novel structures are remarkable for use in the development of highly efficient LEDs. The PL signals of the Cu-doped CdS QDs showed Forster resonance energy transfer (FRET) mediated from the QDs to Cu ions. At this stage, the LED efficiency remains the result of a positively charged surface due to the Cu doping. The existing literature substantiates the argument of correlating fluorescent intensity with Cu doping as a function of charged surface [53,54]. However, further investigation in this regard remains an attractive research pursuit. 

## Data Availability

The study did not report any data.
